# Longitudinal trajectories of total, cognitive-affective, and somatic depressive symptoms in relation to hip fracture risk: evidence from the HRS and ELSA cohorts

**DOI:** 10.3389/fpubh.2026.1845879

**Published:** 2026-05-26

**Authors:** Hongzhou Zhao, Jiange Chen, Jiahui Xing

**Affiliations:** 1 Department of Orthopedics and Traumatology of Integrated Traditional Chinese and Western Medicine, Tianjin Hospital, Tianjin University, Tianjin, China; 2 Department of Orthopedics, First Teaching Hospital of Tianjin University of Traditional Chinese Medicine, Tianjin, China; 3 National Clinical Research Center for Chinese Medicine, Tianjin, China

**Keywords:** cognitive-affective symptom, cohort study, depressive symptom trajectory, dynamic trajectory, hip fracture, somatic symptom

## Abstract

**Background:**

Prior research has established a link between depressive symptoms and the incidence of hip fractures, with most studies relying on a single assessment and failing to differentiate among symptom subtypes. This study utilized repeated measurements to construct symptom trajectories and separately examined the cognitive-emotional and physical dimensions to elucidate their relationship with the risk of hip fractures.

**Methods:**

We analyzed data from individuals aged 45 and older participating in the Health and Retirement Study (HRS, USA) and the English Longitudinal Study of Ageing (ELSA, UK), excluding those with a history of hip fractures. Depressive symptoms were assessed biennially using the Center for Epidemiological Studies Depression Scale (CES-D) over a span of eight consecutive years. Participants were categorized into five trajectory groups based on their CES-D scores: consistently low, decreasing, fluctuating, increasing, and consistently high. Over the subsequent 10 years, the incidence of hip fractures was ascertained through self-reported physician diagnoses. The Cox proportional hazards model was employed to calculate the hazard ratio (HR) and 95% confidence interval (CI) to examine the association between the trajectory of depressive symptoms and the risk of hip fractures, while controlling for sociodemographic factors, health behaviors, and overall health status.

**Results:**

Among 7,014 participants (mean age 64.4 years, 61.2% female), 382 incident hip fractures occurred during follow-up. In the fully adjusted model, participants with increasing total depressive symptoms (HR = 1.53, 95% CI: 1.12–2.09) and consistently high total depressive symptoms (HR = 1.70, 95% CI: 1.12–2.56) had elevated hip fracture risk compared with those with consistently low symptoms. Decreasing (HR = 0.81, 95% CI: 0.53–1.22) and fluctuating trajectories (HR = 1.13, 95% CI: 0.85–1.51) were not significantly associated with hip fracture risk. Regarding symptom subtypes, consistently high cognitive-affective symptoms were associated with increased hip fracture risk (HR = 1.71, 95% CI: 1.09–2.69). For somatic symptoms, both increasing (HR = 1.61, 95% CI: 1.17–2.20) and consistently high trajectories (HR = 1.72, 95% CI: 1.06–2.81) were associated with increased hip fracture risk.

**Conclusion:**

Elevated hip fracture risk is linked to increasing and consistently high total depressive symptom trajectories, whereas decreasing and fluctuating patterns do not significantly differ from consistently low levels. Greater fracture risk is indicated by consistently high cognitive-affective symptoms and both increasing and consistently high somatic symptoms. These findings imply that monitoring depressive symptom trajectories and their subtypes may help identify individuals at elevated fracture risk and inform targeted prevention strategies. Additional research on underlying mechanisms and approaches to identify and address high-risk populations is necessary.

## Introduction

Global population aging has rapidly increased in recent years, with individuals aged 60 and above now accounting for more than 11% of the global population, a proportion projected to double by 2050 (1). This demographic transition has led to a surge in fracture rates, particularly fragility fractures, with hip fractures being a significant concern among older individuals. Data derived from the China Health and Retirement Longitudinal Study revealed that the incidence of hip fractures among middle-aged and older adults rose to 5.78% between 2013 and 2020 (2), with a higher prevalence among women compared to men (3). The World Health Organization anticipates that the annual number of hip fracture cases worldwide will escalate to 6 million by 2050 (4). These fractures result in substantial repercussions such as elevated healthcare expenses (5), prolonged functional impairment (6), and heightened mortality rates (7). Studies indicate that approximately 20–30% of patients succumb within a year post-hip fracture (8, 9), while nearly half of survivors experience a loss of independence in daily activities, with around 30% necessitating long-term care (10). This escalating burden presents significant challenges to global public health systems.

Depression impacts more than one-third of older adults globally (11), posing a significant public health issue. Around 30% of men and 40% of women aged 45 and above encounter depressive symptoms (12), which are linked to various adverse consequences such as cardiovascular disease (13), cognitive decline (14), and functional impairment (15). Moreover, depression is recognized as a contributing factor to hip fracture risk (16). The association can be elucidated through several mechanisms, including the acceleration of bone loss due to imbalances in bone formation and resorption induced by depressive symptoms, as confirmed by a meta-analysis (17). Furthermore, falls contribute to over 95% of hip fractures among older adults (8, 18), with research consistently establishing a connection between depressive symptoms and an elevated risk of falls (19, 20). Given the critical nature of both depression and hip fractures in aging populations, comprehending their interrelation is paramount.

Prior studies have primarily focused on either the presence or absence of depressive symptoms (21–24) or short-term persistent symptoms (≤4 years) (25) concerning hip fracture, neglecting the longitudinal variations in symptom patterns (26, 27). However, research indicates that depressive symptoms exhibit fluctuations over time, characterized by remission, relapse, chronicity, transient elevations due to stressors, and relapse post-initial improvement. Failing to capture these temporal dynamics may lead to an incomplete characterization of the depression-hip fracture association or an oversimplified interpretation of symptom dynamics. Additionally, depressive symptoms comprise distinct cognitive-affective and somatic dimensions (28). No previous studies on depression and traumatic conditions have explored how these dimensions individually relate to the risk of hip fracture. Analyzing the evolution of cognitive-affective and somatic symptoms over time and their respective connections to hip fracture may provide a more nuanced understanding of their associations with hip fracture risk.

This study fills these gaps by delineating enduring trajectories of total, cognitive-affective, and somatic depressive symptoms and investigating their links to the risk of hip fracture. Leveraging information from the Health and Retirement Study (HRS) and the English Longitudinal Study of Ageing (ELSA), we scrutinized 8-year patterns of symptoms to forecast the occurrence of hip fractures over a subsequent 10-year period. Our hypothesis posited that escalating and consistently elevated trajectories would correlate with heightened hip fracture susceptibility, whereas diminishing and irregular trajectories would exhibit no notable correlation.

## Methods

### Study population

Data were obtained from the HRS and ELSA, two nationally representative longitudinal cohorts of aging populations in the United States and the United Kingdom. Both cohorts conduct biennial interviews using standardized questionnaires and comparable instruments to evaluate physical health, mental health, and socioeconomic status. The harmonized procedures enable cross-national analysis of the association between depressive symptom trajectories and the risk of hip fracture.

**Figure 1:**
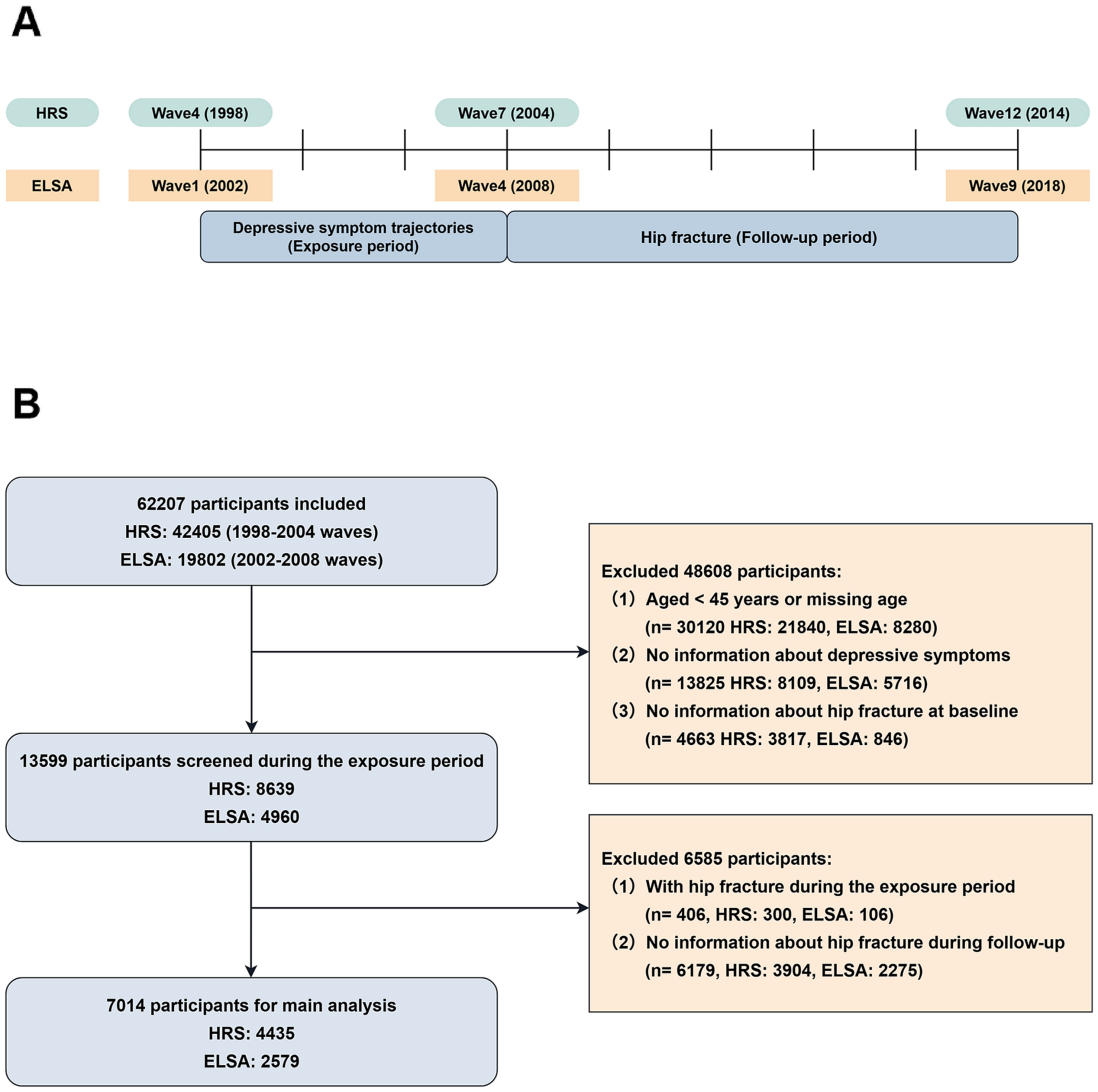
Timeline and sample selection flow of the present study. **(A)** Current study timeline. The exposure period was defined as Waves 4 to 7 of HRS and Waves 1 to 4 of ELSA, whereas the follow-up period included Waves 8 to 12 of HRS and Waves 5 to 9 of ELSA. **(B)** Process for selecting samples in HRS and ELSA according to inclusion and exclusion criteria.

Data from HRS waves 4–12 (1998–2014) and ELSA waves 1–9 (2002–2018) were utilized to investigate the relationship between trajectories of depressive symptoms and the occurrence of hip fractures. The baseline for this analysis was set at HRS wave 4 (1998) and ELSA wave 1 (2002), with subsequent waves up to wave 7 in HRS and wave 4 in ELSA considered as the exposure period. Trajectories were established based on data from four consecutive waves starting from the baseline. The follow-up period, during which hip fracture outcomes were determined, encompassed HRS waves 8–12 and ELSA waves 5–9. A visual representation of the study timeline can be found in Figure 1A.

### Exclusion criteria

Participants under 45 years, with a history of hip fracture before or during the exposure period, incomplete depressive symptom data during the exposure period, or missing hip fracture information during follow-up were excluded. The age threshold of 45 years corresponds to the World Health Organization’s classification of middle-aged and older adults, emphasizing a group more vulnerable to age-related changes that could heighten the risk of both depressive symptoms and hip fractures. The exclusion of individuals with prior or exposure-period hip fractures facilitated a clearer evaluation of the relationship between symptom trajectories and new fractures. The omission of those with inadequate trajectory or outcome data ensured that multiple imputation was exclusively applied to covariates, reducing potential bias in the primary findings. Figure 1B depicts the sample selection process.

### Primary exposures

Depressive symptoms in older populations were assessed using an 8-item version of the Center for Epidemiologic Studies Depression Scale (CES-D) (29, 30). Participants reported the presence of eight specific symptoms over the past week at biennial evaluations. Scores ranged from 0 to 8, with two items phrased positively and reverse-coded (29, 31). The items assessed were: (1) “Felt depressed”; (2) “Everything was an effort”; (3) “Sleep was restless”; (4) “I was happy”; (5) “Felt lonely”; (6) “I enjoyed life”; (7) “Felt sad”; and (8) “Could not get going.” Consistent with previous studies, scores equal to or greater than 3 were considered indicative of clinically significant symptoms (32, 33).

Items were classified into cognitive-affective and somatic subtypes. The cognitive-affective subtype included “Felt depressed,” “Felt lonely,” and “Felt sad,” along with negative responses to “I was happy” and “I enjoyed life.” The somatic subtype consisted of “Everything was an effort,” “Sleep was restless,” and “Could not get going” (34, 35). This two-factor structure has been confirmed through confirmatory factor analysis, demonstrating satisfactory discriminant validity (36). Scores for each domain were dichotomized at the upper tertile, where scores ≥2 indicated significant symptoms (37, 38).

To capture the temporal dynamics of depressive symptoms across four successive assessments (HRS waves 4–7 and ELSA waves 1–4), we categorized participants into five distinct trajectories: consistently low, decreasing, fluctuating, increasing, and consistently high. While formal data-driven modeling (e.g., latent class growth analysis) is frequently utilized in such contexts, these methods often identify subpopulations that primarily reflect parallel gradients of stable severity. To better isolate genuine clinical shifts—such as incident symptom onset or sustained remission—we intentionally adopted a predefined, rule-based classification approach. Anchored to validated clinical cutoffs, this method provides explicit clinical interpretability and maximizes cross-study reproducibility (32, 39).

To ensure strict operational reproducibility and eliminate descriptive ambiguity, the five mutually exclusive trajectories were operationalized using a deterministic binary algorithm. At each wave, participants were classified as (+) if their score met or exceeded the clinical threshold (≥ 3 for total symptoms) and (−) if it remained below the threshold. As exhaustively mapped in Supplementary Table 1, the consistently low trajectory comprised individuals scoring (−) at all four assessments, whereas the consistently high trajectory included those consistently scoring (+). The decreasing trajectory was strictly defined by an initial depressed state followed by sustained remission (transitioning from + to - without subsequent rebound). Conversely, the increasing trajectory captured an initial non-depressed state transitioning to sustained depression (transitioning from - to + without subsequent remission). The fluctuating trajectory encompassed all remaining irregular patterns that did not meet the aforementioned criteria. The consistently low group served as the reference. Trajectories of somatic and cognitive-affective symptoms were similarly operationalized using a respective domain threshold of ≥ 2.

### Hip fracture outcomes

Incident hip fractures were identified based on data from HRS waves 8–12 and ELSA waves 5–9. Hip fracture status in both cohorts was ascertained through self-report or proxy report of physician diagnosis in response to the question: “Has a doctor ever informed you of a hip fracture?” Participants indicating a hip fracture at any follow-up wave were categorized as having experienced an event. In cases where a fracture was reported in the previous wave, a validation question was posed in the subsequent wave; if the participant denied the earlier report, the hip fracture status was rectified retroactively. Previous studies have shown the reliability of self-reported hip fractures to be high when compared with hospital records.

### Covariates

**Figure 2:**
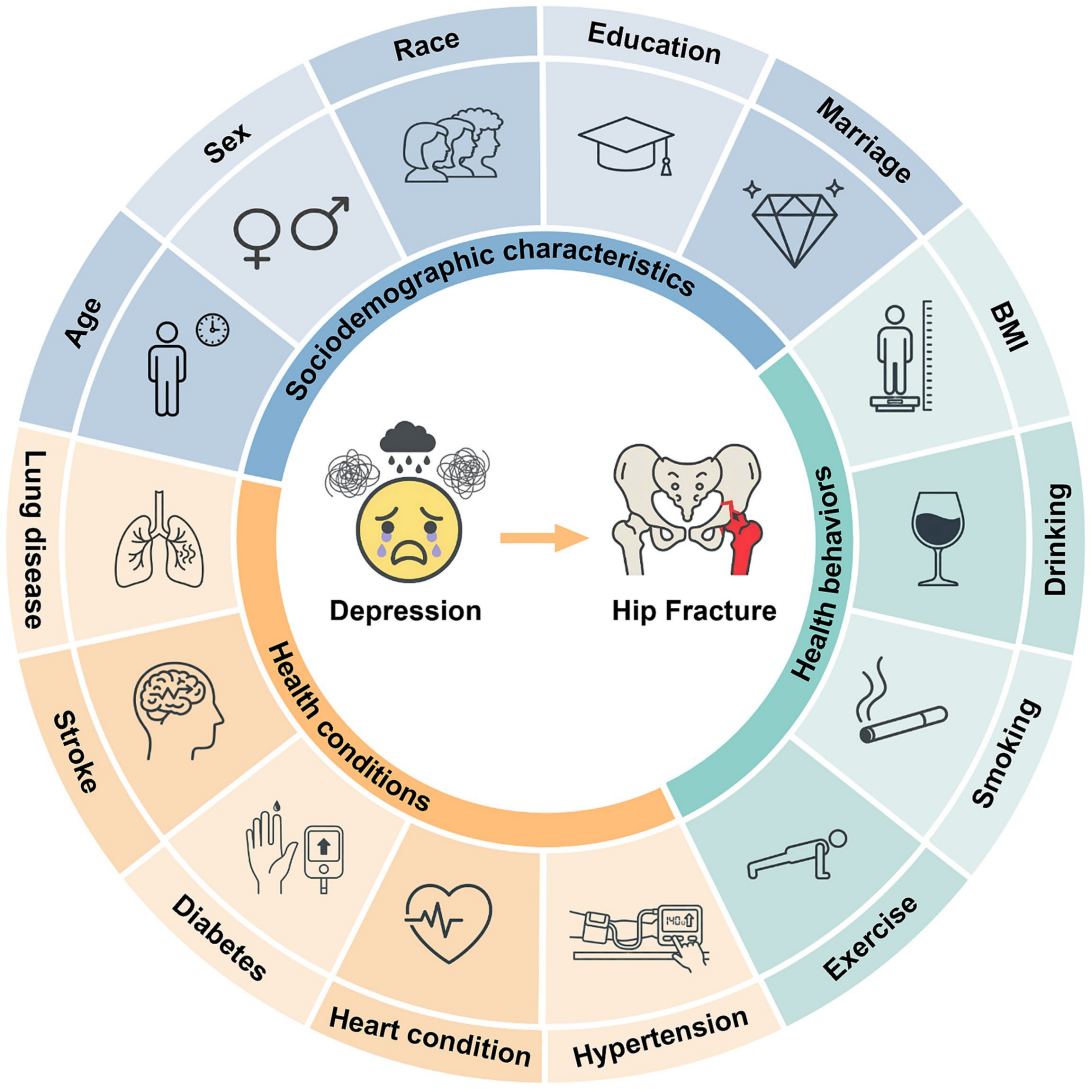
Illustrating covariate classification employed in this study. The main covariates include three categories: sociodemographic characteristics, health behaviors, and health conditions.

Covariates were chosen to address potential confounding in the association between depressive symptoms and the risk of hip fracture. Baseline sociodemographic variables comprised age (continuous), sex, race (White or non-White), education level (high school or lower, some college, college or higher), and marital status (married/partnered or separated/divorced/widowed/never married). Baseline health behaviors included body mass index (BMI, continuous), alcohol consumption (never or ever), smoking status (never or ever), and frequency of vigorous physical activity (more than once a week, once a week or 1–3 times a month, never). Self-reported physician-diagnosed conditions encompassed hypertension, heart disease, diabetes, stroke, and lung disease. Previous research has demonstrated substantial concordance between self-reported health conditions in HRS and medical records, with robust external validity for behavioral assessments (40, 41). The categorization of covariates is illustrated in Figure 2.

Notably, while antidepressant use is a potential risk factor for hip fracture, it was not included as a covariate in our primary models due to significant data constraints. Specifically, the HRS only introduced psychiatric medication indicators from Wave 8 onwards, which were subject to substantial missing values and lacked pharmacological specificity. Similarly, the ELSA database only began recording psychiatric medication in its most recent two waves, providing insufficient longitudinal data to cover our 8-year exposure period. Consequently, antidepressant use could not be robustly harmonized or controlled for across all assessment waves.

### Statistical analysis

Baseline demographic characteristics were analyzed descriptively, categorized by depressive symptom trajectory. Continuous variables were presented as means with standard deviations (SD) or medians with interquartile ranges (IQR), while categorical variables were expressed as frequencies with percentages. Time was defined as the duration from the commencement of follow-up to the occurrence of the first hip fracture or the conclusion of follow-up, whichever came first. Follow-up was terminated at the time of the participant’s last completed survey. The proportional hazards assumption was evaluated using the Schoenfeld residuals test, confirming its validity by observing no correlation between residuals and time. Cox proportional hazards models were utilized to calculate hazard ratios (HRs) and 95% confidence intervals (CIs) to assess the relationship between depressive symptom trajectories and the risk of hip fracture. The consistently low trajectory served as the reference group in four models: (1) Model 1, adjusted for trajectory and age; (2) Model 2, further adjusted for sociodemographic variables (sex, race, education, marital status); (3) Model 3, additionally adjusted for health behaviors (BMI, alcohol consumption, smoking, physical activity); (4) Model 4, additionally adjusted for health conditions (hypertension, heart disease, diabetes, stroke, and lung disease). Missing covariate data were addressed using multiple imputation by chained equations (MICE) in R. Five imputed datasets were generated, with five iterations performed for each imputation procedure. All statistical models were fitted separately within each imputed dataset, and the resulting estimates were combined using Rubin’s rules to account for both within- and between-imputation variability.

Additional and sensitivity analyses were conducted to evaluate the robustness of the findings and address potential sources of bias. First, we repeated the main Cox proportional hazards models using more stringent definitions of the decreasing and increasing trajectories, excluding participants whose scores crossed the symptom threshold only at the final exposure wave. Second, we re-estimated all four Cox models with additional adjustment for income, cancer, and arthritis. Third, because hip fracture events were assessed at discrete survey waves rather than exact event dates, we fitted discrete-time complementary log–log survival models (42) corresponding to Models 3 and 4. Fourth, to compare trajectory-based findings with single-time-point depressive symptom status, we re-estimated Models 3 and 4 using logistic regression. In addition, to assess potential selection bias, we conducted an attrition analysis comparing baseline characteristics between participants included in the analytical sample and those excluded because of incomplete data. Because death may preclude the occurrence of hip fracture in older adults, we also performed a competing risk analysis using Fine-Gray subdistribution hazard models, treating death before hip fracture as a competing event and estimating subdistribution hazard ratios (sdHRs). This analysis incorporated death as a competing event and retained participants with ascertainable hip fracture or death status; therefore, the analytic sample differed from that used in the primary Cox models. Finally, to examine potential effect modification, we conducted subgroup analyses of total depressive symptom trajectories stratified by sex and age (<65 vs. ≥65 years) and tested multiplicative interaction terms in the fully adjusted Fine-Gray models.

All analyses were performed using R software (version 4.5.2). Two-sided tests were conducted, with *p* < 0.05 considered statistically significant.

## Results

An attrition analysis revealed systematic differences between the included cohort and those excluded due to missing data (Supplementary Table 6). Excluded participants were generally older, had a higher prevalence of chronic conditions such as hypertension and diabetes, and exhibited higher baseline CES-D scores (all *p* < 0.001).

Following the application of exclusion criteria, 7,014 participants from the two cohorts were analyzed. During the 10-year follow-up period, 382 new hip fractures were documented. Our investigation revealed that various depressive symptom trajectories were linked to the incidence of hip fractures, with different subtypes and trajectories demonstrating unique associations with fracture susceptibility.

Tables 1, 2 display the baseline sociodemographic characteristics, health behaviors, and health conditions of the combined sample categorized by total depressive symptom trajectory. The average age at baseline was 64.4 years. The majority of participants were female (61.2%), White (89.8%), and had a high school education or lower (60.3%). The mean BMI was 27.4 kg/m^2^. Of the participants, 55.2% had a history of smoking, 67.3% had consumed alcohol at some point, and 56.5% had never participated in vigorous physical activity. Throughout the observation period, 62.9% of the participants were assigned to the consistently low trajectory group and were more likely to be married or in a partnership (77.2%). Conversely, individuals in the consistently high trajectory group were predominantly those with a high school education or less (84.4%) and exhibited a higher prevalence of chronic conditions: 17.9% had heart disease, 5.6% had experienced a stroke, 5.6% had lung disease, 46.8% had hypertension, and 11.0% had diabetes.

**Table 1:** Initial characteristics of study participants at the beginning of the exposure period.

Variables	Overall sample	Depressive symptoms trajectory group				
		Consistently low	Decreasing	Fluctuating	Increasing	Consistently high
Individuals, *n* (%)	7,014	4,412 (62.9)	609 (8.7)	1,079(15.4)	613(8.7)	301(4.3)
Sociodemographic characteristics						
Age, y, mean (SD)	64.4 (6.2)	64.3 (6.1)	64.0 (6.4)	64.3 (6.2)	65.1 (6.4)	64.6 (6.5)
Sex, *n* (%)						
Female	4,292 (61.2)	2,417 (54.8)	422 (69.3)	761 (70.5)	456 (74.4)	236 (78.4)
Male	2,722 (38.8)	1995 (45.2)	187 (30.7)	318 (29.5)	157 (25.6)	65 (21.6)
Race, *n* (%)						
White	6,302 (89.8)	4,052 (91.8)	530 (87.0)	940 (87.1)	538 (87.8)	242 (80.4)
Non-white	712 (10.2)	360 (8.2)	79 (13.0)	139 (12.9)	75 (12.2)	59 (19.6)
Highest degree in education, *n* (%)						
High school and below	4,231 (60.3)	2,433 (55.1)	411 (67.5)	740 (68.6)	393 (64.1)	254 (84.4)
Some college	1,446 (20.6)	989 (22.4)	103 (16.9)	188 (17.4)	133 (21.7)	33 (11.0)
College and above	1,337 (19.1)	990 (22.4)	95 (15.6)	151 (14.0)	87 (14.2)	14 (4.7)
Marital status, *n* (%)						
Married or partnered	5,055 (72.1)	3,407 (77.2)	374 (61.4)	715 (66.3)	411 (67.0)	148 (49.2)
Separated/ Divorced/ Widowed/ Never married	1959 (27.9)	1,005 (22.8)	235 (38.6)	364 (33.7)	202 (33.0)	153 (50.8)

Number of missing values before imputation: None.

**Table 2:** Initial health behaviors and conditions for analytical sample at the beginning of exposure period.

Variables	Overall sample	Depressive symptoms trajectory group				
		Consistently low	Decreasing	Fluctuating	Increasing	Consistently high
Individuals, n (%)	7,014	4,412 (62.9)	609 (8.7)	1,079(15.4)	613(8.7)	301(4.3)
Health behaviors						
BMI, kg/m^2^, mean (SD)	27.4 (4.7)	27.1 (4.4)	28.0 (5.0)	27.7 (5.0)	28.1 (4.9)	29.1 (6.1)
Drinking status, *n* (%)						
Never drinkers	2,292 (32.7)	1,279 (29.0)	222 (36.5)	395 (36.6)	239 (39.0)	157 (52.2)
Ever drinkers	4,722 (67.3)	3,133 (71.0)	387 (63.5)	684 (63.4)	374 (61.0)	144 (47.8)
Smoking status, *n* (%)						
Never smokers	3,131 (44.8)	1998 (45.5)	269 (44.4)	483 (44.9)	265 (43.4)	116 (38.5)
Ever smokers	3,858 (55.2)	2,398 (54.5)	337 (55.6)	592 (55.1)	346 (56.6)	185 (61.5)
Vigorous exercise, *n* (%)						
More than once per week	1742 (24.8)	1,262 (28.6)	130 (21.3)	219 (20.3)	103 (16.8)	28 (9.3)
Once per week or 1–3 times per month	1,312 (18.7)	897 (20.3)	93 (15.3)	179 (16.6)	106 (17.3)	37 (12.3)
Never	3,960 (56.5)	2,253 (51.1)	386 (63.4)	681 (63.1)	404 (65.9)	236 (78.4)
Health conditions						
Heart condition (yes/no), *n* (%)	804 (11.5)	461 (10.4)	72 (11.8)	143 (13.3)	74 (12.1)	54 (17.9)
Stroke (yes/no), *n* (%)	169 (2.4)	83 (1.9)	20 (3.3)	31 (2.9)	18 (2.9)	17 (5.6)
Lung disease (yes/no), *n* (%)	211 (3.0)	103 (2.3)	39 (6.4)	37 (3.4)	15 (2.4)	17 (5.6)
Hypertension (yes/no), *n* (%)	2,499 (35.6)	1,467 (33.3)	238 (39.1)	404 (37.4)	249 (40.6)	141 (46.8)
Diabetes (yes/no), *n* (%)	501 (7.1)	264 (6.0)	50 (8.2)	93 (8.6)	61 (10.0)	33 (11.0)

BMI stands for body mass index. The percentage of missing values before imputation is as follows: BMI 3.49%, Smoking 0.36%. There are no missing values for the other variables.

**Figure 3:**
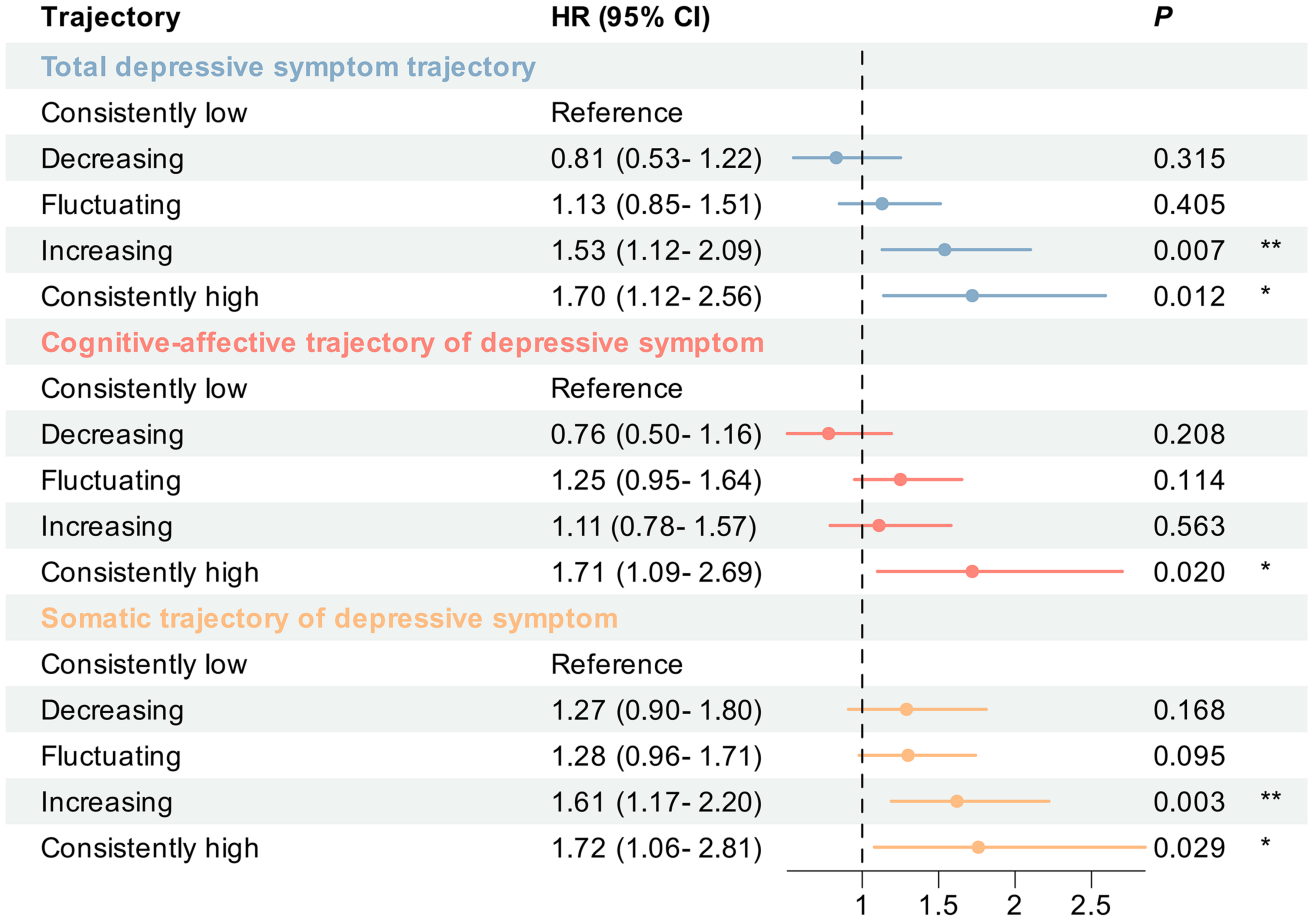
Fully adjusted associations between depressive symptom trajectories and hip fracture risk. Forest plot showing hazard ratios (HRs) and 95% confidence intervals (CIs) for the associations of total, cognitive-affective, and somatic depressive symptom trajectories with incident hip fracture in the fully adjusted Cox proportional hazards model. The consistently low trajectory served as the reference group. The fully adjusted model included age, sex, race, education, marital status, body mass index, alcohol consumption, smoking status, vigorous exercise, hypertension, heart condition, diabetes, stroke, and lung disease. The vertical dashed line indicates the null value (HR = 1). Significant associations are indicated by asterisks (^*^p < 0.05; ^**^p < 0.01).

Table 3 presents the associations between total, cognitive-affective, and somatic depressive symptom trajectories and the risk of hip fracture in the combined sample across progressively adjusted Cox models. The fully adjusted estimates from Model 4 are visualized in Figure 3, whereas the estimates from Models 1–3 are provided in Supplementary Figure 1. In Model 4, after adjustment for sociodemographic characteristics, health behaviors, and health conditions, participants with increasing total depressive symptoms (HR = 1.53, 95% CI: 1.12–2.09) had a higher risk of hip fracture than those with consistently low symptoms. A consistently high total depressive symptom trajectory was also associated with an elevated risk of hip fracture (HR = 1.70, 95% CI: 1.12–2.56). In contrast, the decreasing (HR = 0.81, 95% CI: 0.53–1.22) and fluctuating trajectories (HR = 1.13, 95% CI: 0.85–1.51) were not significantly associated with hip fracture risk.

**Table 3:** Cox proportional hazard ratios examining the relationship between depressive symptom trajectories and hip fracture over a decade for the whole sample.

Depressive symptoms trajectory group	No. of cases (%)	Model 1 ^†^		Model 2 ^‡^		Model 3 ^§^		Model 4 ^¶^	
		HR (95% CI)	*p*	HR (95% CI)	*p*	HR (95% CI)	*p*	HR (95% CI)	*p*
Total depressive symptom trajectory									
Consistently low	4,412 (62.9)	Reference		Reference		Reference		Reference	
Decreasing	609 (8.7)	0.85 (0.57–1.28)	0.437	0.83 (0.55–1.25)	0.377	0.83 (0.55–1.26)	0.385	0.81 (0.53–1.22)	0.315
Fluctuating	1,079 (15.4)	1.17 (0.89–1.56)	0.263	1.15 (0.87–1.53)	0.330	1.14 (0.86–1.52)	0.364	1.13 (0.85–1.51)	0.405
Increasing	613 (8.7)	1.60 (1.18–2.18)	0.002^**^	1.56 (1.14–2.12)	0.005^**^	1.56 (1.15–2.13)	0.005^**^	1.53 (1.12–2.09)	0.007^**^
Consistently high	301 (4.3)	1.81 (1.22–2.69)	0.003^**^	1.78 (1.19–2.68)	0.005^**^	1.72 (1.14–2.59)	0.010^*^	1.70 (1.12–2.56)	0.012^*^
Cognitive-affective trajectory of depressive symptom									
Consistently low	4,617 (65.8)	Reference		Reference		Reference		Reference	
Decreasing	599 (8.5)	0.82 (0.54–1.24)	0.336	0.79 (0.52–1.20)	0.263	0.78 (0.51–1.19)	0.244	0.76 (0.50–1.16)	0.208
Fluctuating	1,002 (14.3)	1.32 (1.01–1.73)	0.046^*^	1.27 (0.97–1.67)	0.084	1.25 (0.95–1.65)	0.108	1.25 (0.95–1.64)	0.114
Increasing	581 (8.3)	1.18 (0.84–1.67)	0.337	1.14 (0.80–1.61)	0.462	1.13 (0.80–1.60)	0.490	1.11 (0.78–1.57)	0.563
Consistently high	215 (3.1)	1.87 (1.21–2.91)	0.005^**^	1.83 (1.17–2.87)	0.009^**^	1.73 (1.10–2.71)	0.018^*^	1.71 (1.09–2.69)	0.020^*^
Somatic trajectory of depressive symptom									
Consistently low	4,475 (63.8)	Reference		Reference		Reference		Reference	
Decreasing	677 (9.6)	1.33 (0.95–1.87)	0.098	1.33 (0.94–1.87)	0.102	1.30 (0.92–1.84)	0.131	1.27 (0.90–1.80)	0.168
Fluctuating	1,014 (14.5)	1.32 (0.99–1.75)	0.057	1.31 (0.98–1.74)	0.064	1.30 (0.97–1.73)	0.075	1.28 (0.96–1.71)	0.095
Increasing	618 (8.8)	1.64 (1.21–2.23)	0.002^**^	1.61 (1.18–2.20)	0.003^**^	1.62 (1.18–2.21)	0.003^**^	1.61 (1.17–2.20)	0.003^**^
Consistently high	230 (3.3)	1.80 (1.13–2.89)	0.014^*^	1.79 (1.11–2.88)	0.018^*^	1.78 (1.10–2.89)	0.020^*^	1.72 (1.06–2.81)	0.029^*^

^†^Model 1 adjusts for age only. ^‡^Model 2 additionally adjusts for sociodemographics (sex, race, education, and marital status). ^§^Model 3 additionally adjusts for health behaviors (body mass index, alcohol consumption, smoking status, and vigorous exercise). ^¶^Model 4 additionally adjusts for health conditions (hypertension, heart condition, diabetes, stroke, and lung disease). Significant associations are indicated by asterisks (^*^p < 0.05; ^**^p < 0.01).

When depressive symptoms were divided into cognitive-affective and somatic subtypes, different patterns of association were observed. In the fully adjusted model, participants with a consistently high cognitive-affective symptom trajectory had a significantly higher risk of hip fracture than those with consistently low cognitive-affective symptoms (HR = 1.71, 95% CI: 1.09–2.69). The decreasing (HR = 0.76, 95% CI: 0.50–1.16), fluctuating (HR = 1.25, 95% CI: 0.95–1.64), and increasing trajectories (HR = 1.11, 95% CI: 0.78–1.57) were not significantly associated with hip fracture risk.

For somatic symptoms, both the increasing and consistently high trajectories were associated with elevated hip fracture risk in the fully adjusted model. Compared with the consistently low trajectory, the HR was 1.61 (95% CI: 1.17–2.20) for the increasing somatic trajectory and 1.72 (95% CI: 1.06–2.81) for the consistently high somatic trajectory. The decreasing (HR = 1.27, 95% CI: 0.90–1.80) and fluctuating somatic trajectories (HR = 1.28, 95% CI: 0.96–1.71) were not significantly associated with hip fracture risk.

The sensitivity analyses generally supported the robustness of the main findings (Supplementary Tables 2–5). The associations of increasing and consistently high total depressive symptom trajectories with elevated hip fracture risk remained broadly consistent after additional covariate adjustment, stricter trajectory definitions, discrete-time survival modeling, and logistic regression. The Fine-Gray competing risk analysis also yielded results broadly comparable with the primary Cox models (Supplementary Table 7). Subgroup analyses of total depressive symptom trajectories showed generally similar patterns across sex and age categories (Supplementary Tables 8, 9). There was no consistent evidence of effect modification by sex or age for the main risk-elevating trajectories, although one interaction term for the decreasing trajectory by age reached statistical significance.

## Discussion

To our knowledge, this is among the first studies to conduct a pooled analysis of nationally representative cohorts from the United States and the United Kingdom to investigate the relationship between long-term depressive symptom trajectories and hip fracture risk. Based on predefined scoring criteria across four waves, we identified five distinct trajectories: consistently low, decreasing, fluctuating, increasing, and consistently high. Neither decreasing nor fluctuating trajectories for total, cognitive-affective, and somatic depressive symptoms exhibited significant associations with hip fracture risk. Conversely, increasing and consistently high trajectories of total depressive symptoms, consistently high trajectories of cognitive-affective symptoms, and increasing and consistently high trajectories of somatic symptoms were associated with higher hip fracture risk. These associations remained significant even after adjusting for multiple potential confounders. Sensitivity analyses, which considered variations in analytical models, trajectory definitions, and covariate adjustments, generally supported the robustness of our findings. This study further extends previous work by examining trajectories of cognitive-affective and somatic depressive symptoms and the risk of hip fracture.

An investigation utilizing data from the China Health and Retirement Longitudinal Study revealed that individuals exhibiting high baseline depressive symptoms faced an elevated risk of hip fracture during a 7-year monitoring period (21). Two meta-analyses indicated that depression or depressive symptoms correlated with a 20–30% increase in the likelihood of hip fractures (22, 24). A study based on Taiwan’s National Health Insurance database demonstrated that individuals with depression had a 1.34-fold higher risk of hip fractures compared to their age-matched counterparts (43). Previous research suggested a direct correlation between the severity of depressive symptoms and the risk of hip fractures, with only major depression significantly heightening the risk. However, these conclusions were drawn from singular baseline evaluations, which could lead to inaccuracies in determining depressive status. Given the dynamic and fluctuating nature of depressive symptoms, continuous long-term assessments are essential to precisely capture developmental patterns and gain deeper insights into their relationship with hip fracture susceptibility. A single time point fails to adequately represent the intricate and evolving characteristics of depressive symptoms. While some earlier studies explored changes over two consecutive follow-up periods (25, 44), the limited data points restricted these analyses to capturing only a fraction of the temporal variations. For individuals with fluctuating symptoms, differences between two time points are inadequate to depict extended patterns accurately. Studies often categorized symptoms as low-stable, moderate-stable, or high-increasing trajectories. Their results consistently showed that only those with high-increasing trajectories had a significantly higher risk of hip fractures compared to those with low-stable trajectories; moderate-stable trajectories did not exhibit this heightened risk. We contend that these classifications primarily represent symptom severity rather than genuine dynamic trajectories. Our findings indicate that persistent depressive symptoms, whether gradually escalating or consistently severe, correlate with an increased risk of hip fractures compared to consistently low trajectories. Individuals with prolonged symptom relief may not face an elevated risk of hip fractures, even if they previously experienced severe symptoms. This raises the possibility that effective management of depressive symptoms may be relevant to fracture-risk reduction, although this hypothesis requires confirmation in interventional studies. To delve deeper into how trajectories of depressive symptoms are associated with the risk of hip fractures, we further divided total symptoms into cognitive-affective and somatic subcategories to explore the underlying mechanisms.

Previous research has suggested two primary pathways linking depressive symptoms to the risk of hip fractures: decreased bone mineral density and increased susceptibility to falls (44). For the cognitive-affective subtype, which is characterized by emotional distress and anxiety, the underlying mechanisms may largely involve the psychological stress response. Chronic emotional stress has been widely recognized to dysregulate the hypothalamic–pituitary–adrenal (HPA) axis, potentially leading to elevated circulating cortisol (45–47). Prolonged exposure to elevated glucocorticoids can negatively affect bone remodeling by suppressing bone formation and promoting bone resorption (48). In addition to endocrine dysregulation, persistent psychological stress is frequently accompanied by low-grade systemic inflammation, another known contributor to bone deterioration (49–51). Our observation that a consistently high cognitive-affective trajectory relates to increased fracture risk suggests that enduring, unresolved psychological burden might chronically activate these adverse physiological pathways.

In comparison, somatic symptoms—such as fatigue, sleep disturbances, and psychomotor retardation—demonstrated a robust predictive capacity for hip fractures, which may be more directly tied to biomechanical vulnerabilities. Psychomotor retardation often presents clinically as compromised postural control and altered gait dynamics, factors that are known to substantially increase the likelihood of falls during daily activities (52). Furthermore, chronic sleep disruptions can impair daytime attention and motor coordination, directly elevating fall susceptibility (53, 54). Beyond fall risk, poor sleep quality has also been linked to reduced bone mineral density, possibly by altering normal bone turnover processes (55, 56). The elevated fracture risk observed in trajectories with increasing or consistently high somatic symptoms could reflect the cumulative effects of prolonged exposure to these physical and biomechanical impairments.

The two subtypes of depression are closely linked and can collectively establish a harmful cycle that heightens the risk of hip fractures. Research indicates that anxiety, a fundamental aspect of cognitive-affective symptoms, significantly heightens the fear of falling (57). This fear can trigger a cautious style of walking (53), characterized by shorter steps and slower pace, a gait pattern that paradoxically increases instability and further elevates the risk of falls. This indicates that cognitive-affective conditions might indirectly exacerbate the biomechanical risks associated with the somatic subtype. Furthermore, depressive symptoms can result in nutritional deficiencies, such as decreased appetite leading to insufficient intake of calcium and vitamin D (58). Depression is also linked to a higher prevalence of smoking, which is well-established to harm bone health through various mechanisms, including disruptions in sex hormone levels and increased oxidative stress (58). The use of antidepressants is another factor that must be considered when analyzing the connection between depressive symptoms and fractures. Several studies have shown that selective serotonin reuptake inhibitors (SSRIs) are linked to lower bone mineral density and an increased risk of fractures (58), possibly through direct adverse effects on bone metabolism (59). Nevertheless, some studies have indicated that the relationship between depressive symptoms and fracture risk persists even after excluding participants using antidepressants, suggesting that depressive symptoms may be independently associated with bone health and fracture risk (44).

While these physiological, endocrine, and behavioral pathways offer theoretical explanations for our findings, they must be interpreted with caution. Our analysis relied on observational epidemiological data, and without concurrent assessments of bone turnover markers, inflammatory cytokines, or objective gait metrics, these mechanistic links remain speculative. Future prospective studies incorporating direct biological and biomechanical measurements are needed to confirm the precise pathways mediating the relationship between distinct depressive symptom trajectories and hip fracture risk.

By distinguishing cognitive-affective and somatic depressive symptom subtypes, this study provides exploratory evidence that different symptom dimensions may be associated with hip fracture risk through partly distinct pathways. Our findings align with the hypothesis that reduced bone density and increased fall risk, the main determinants of hip fracture, may correspond to the core features of depressive symptoms. Based on these theoretical pathways, our findings align with the framework that cognitive-affective symptoms may be linked to accelerated bone resorption and systemic bone loss, while somatic symptoms may be linked to increased fall risk. These findings may inform future development of tailored prevention and intervention strategies based on depressive symptom subtype. For patients with predominantly somatic symptoms, clinicians may consider strategies targeting sleep quality, mobility, and fall prevention. For those with predominantly cognitive-affective symptoms, psychological support, stress management, and social connection may be particularly relevant.

While our 8-year trajectory framework provides longitudinal epidemiological evidence for the association between depression and hip fracture, we acknowledge that requiring nearly a decade of observation is challenging to implement in routine clinical practice. However, recent evidence suggests that shorter assessment windows can achieve comparable risk stratification. For example, a recent longitudinal study utilizing the CHARLS cohort demonstrated that evaluating depressive symptoms over a 4-year window (comprising just three biennial assessments) successfully identified a “high-increasing” trajectory that was significantly associated with subsequent hip fracture risk (25). From a clinical perspective, this implies that primary care providers do not need to wait eight years to intervene. Integrating brief, standardized screening tools—such as the short-form CES-D—into routine annual or biennial geriatric health check-ups could be highly pragmatic. By tracking score changes over two to three consecutive visits, clinicians may be able to identify older adults with consistently high or rapidly worsening depressive symptoms. These individuals could then be prioritized for earlier fracture-risk assessment and targeted preventive strategies, including bone mineral density evaluation, fall-prevention education, and appropriate psychological support.

This study possesses multiple strengths. Initially, we integrated data from large, nationally representative prospective cohorts in the United States and the United Kingdom, incorporating repeated assessments of depressive symptoms, extended follow-up periods, and a broad array of covariates considered as potential confounders. Through extensive sensitivity analyses, we further bolstered the generalizability and robustness of our results. Secondly, we differentiated between various depressive symptom subtypes and formulated long-term trajectories for both overall and subtype-specific symptoms. This approach unveiled varying risks of hip fracture across trajectories and offered complementary insights into the association between depression and hip fracture risk. Lastly, as participants were not selected based on mental health status, the sample represents the general population more accurately.

Several limitations should be acknowledged. First, although we used a classification of somatic symptoms that was appropriate for the 8-item CES-D, this definition may not be fully comparable with those used in other studies or depression scales (60). Second, participants with incomplete CES-D data during the exposure period and those who experienced hip fracture during this period were excluded, which may limit the generalizability of our findings to these groups. In addition, because the study population was restricted to adults aged 45 years and older, the findings may not apply to younger populations. Third, depressive symptoms, hip fracture status, and covariates were obtained from self-reported questionnaires, which may introduce recall or reporting bias. Fourth, residual confounding remains possible. We were unable to adjust for several clinically relevant factors, including baseline osteoporosis, fall history, and antidepressant use. Although HRS and ELSA contain some information on osteoporosis and falls, these variables had substantial missingness across the four-wave exposure period. Similarly, detailed antidepressant data were either introduced late or lacked pharmacological specificity, making robust harmonized adjustment across cohorts infeasible. Because these factors may independently influence fracture risk, the observed associations should be interpreted with caution. Fifth, requiring complete depressive symptom data across the four-wave exposure period may have introduced survival and selection bias. As shown in the attrition analysis (Supplementary Table 6), participants excluded because of incomplete data were older, had a greater burden of chronic diseases, and had higher baseline CES-D scores. Therefore, the analytical sample may represent a relatively healthier survivor cohort. If excluded participants had both more severe depressive trajectories and higher fracture risk, the observed associations may underestimate the true magnitude of the relationship between persistent or worsening depressive symptoms and incident hip fracture. Finally, the predefined rule-based trajectory classification has inherent limitations. Although this approach is clinically interpretable and reproducible, dichotomizing continuous CES-D scores may lead to a loss of information on symptom severity. For example, individuals just above the threshold and those with much more severe symptoms are treated as belonging to the same symptom state. In addition, minor fluctuations around the cutoff, potentially reflecting measurement error or transient mood changes, may lead to classification into the fluctuating trajectory even when the underlying depressive state is relatively stable. Future studies using complementary data-driven trajectory models may help validate and refine these findings.

## Conclusion

Our results indicate that maintaining consistently high levels of total depressive symptoms is linked to a higher risk of hip fractures. Conversely, individuals experiencing decreasing or fluctuating depressive symptoms do not exhibit a significantly elevated risk compared to those with consistently low symptoms. Subgroup analysis demonstrates that persistent high levels of cognitive-affective symptoms, as well as increasing or consistently high levels of somatic symptoms, are associated with an increased risk of hip fractures. These findings suggest that repeated assessment of depressive symptom trajectories and their subtypes may help identify individuals at higher fracture risk. Further investigation is necessary to elucidate the potential mechanisms underlying these associations and the dynamic nature of depressive symptom patterns to design more precise interventions for at-risk populations. Given the observational design and potential residual confounding, these findings should be interpreted as associations rather than causal effects.

## Data Availability

The raw data supporting the conclusions of this article will be made available by the authors, without undue reservation.
