# Supplementary material for: Longitudinal trajectories of total, cognitive-affective, and somatic depressive symptoms in relation to hip fracture risk: evidence from the HRS and ELSA cohorts
Source: Front Public Health. 2026 May 26;14:1845879. doi: 10.3389/fpubh.2026.1845879 (PMC13246628; doi:10.3389/fpubh.2026.1845879)
Supplement: Supplementary file 1 [file Data_Sheet_1.docx]

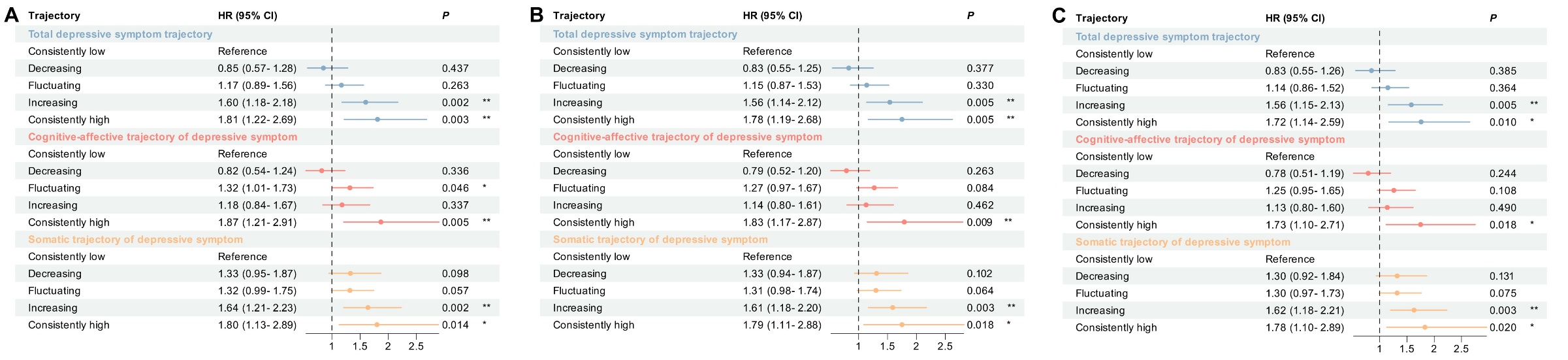


**Supplementary Figure 1.** Associations between depressive symptom trajectories and hip fracture risk across progressively adjusted Cox models. Forest plots showing hazard ratios (HRs) and 95% confidence intervals (CIs) for the associations of total, cognitive-affective, and somatic depressive symptom trajectories with incident hip fracture in Models 1-3. The consistently low trajectory served as the reference group. **Panel** **A** shows Model 1, adjusted for age only. **Panel** **B** shows Model 2, additionally adjusted for sociodemographic characteristics, including sex, race, education, and marital status. **Panel C** shows Model 3, additionally adjusted for health behaviors, including body mass index, alcohol consumption, smoking status, and vigorous exercise. The vertical dashed line indicates the null value (HR = 1). Significant associations are indicated by asterisks (**^*^***P* < 0.05; **^**^***P* < 0.01).

**Supplementary Table 1.** Operational definitions of depressive symptom trajectories

|  | First wave | Second wave | Third wave | Fourth wave |
| --- | --- | --- | --- | --- |
| Consistently low | **-** | **-** | **-** | **-** |
| Consistently high | **+** | **+** | **+** | **+** |
| Decreasing | **+** | **-** | **-** | **-** |
|  | **+** | **+** | **-** | **-** |
|  | **+** | **+** | **+** | **-** |
| Increasing | **-** | **+** | **+** | **+** |
|  | **-** | **-** | **+** | **+** |
|  | **-** | **-** | **-** | **+** |
| Fluctuating | All other cases where the above conditions are not met. | | | |

**Supplementary Table 2.** Cox proportional hazard ratios adjusted for income, arthritis, and cancer as additional covariates

|  |  | **Model 1 ^†^** |  | **Model 2 ^‡^** |  | **Model 3 ^§^** |  | **Model 4 ^¶^** |  |
| --- | --- | --- | --- | --- | --- | --- | --- | --- | --- |
|  | **No. of cases (%)** | **HR (95% CI)** | ***P*** | **HR (95% CI)** | ***P*** | **HR (95% CI)** | ***P*** | **HR (95% CI)** | ***P*** |
| *Total depressive symptom trajectory* | | | | | | | | | |
| Consistently low | 4412 (62.9) | Reference |  | Reference |  | Reference |  | Reference |  |
| Decreasing | 609 (8.7) | 0.85 (0.57- 1.28) | 0.437 | 0.83 (0.55- 1.25) | 0.370 | 0.83 (0.55- 1.26) | 0.378 | 0.79 (0.52- 1.20) | 0.274 |
| Fluctuating | 1079 (15.4) | 1.17 (0.89- 1.56) | 0.263 | 1.15 (0.86- 1.53) | 0.335 | 1.14 (0.85- 1.52) | 0.373 | 1.10 (0.82- 1.47) | 0.518 |
| Increasing | 613 (8.7) | 1.60 (1.18- 2.18) | 0.002**^**^** | 1.56 (1.15- 2.12) | 0.005**^**^** | 1.57 (1.15- 2.14) | 0.005**^**^** | 1.50 (1.10- 2.05) | 0.011**^*^** |
| Consistently high | 301 (4.3) | 1.81 (1.22- 2.69) | 0.003**^**^** | 1.76 (1.17- 2.64) | 0.007**^**^** | 1.69 (1.12- 2.55) | 0.013**^*^** | 1.59 (1.05- 2.42) | 0.028**^*^** |
| *Cognitive-affective trajectory of depressive symptom* | | | | | | | | | |
| Consistently low | 4617 (65.8) | Reference |  | Reference |  | Reference |  | Reference |  |
| Decreasing | 599 (8.5) | 0.82 (0.54- 1.24) | 0.336 | 0.79 (0.52- 1.20) | 0.263 | 0.78 (0.51- 1.18) | 0.242 | 0.75 (0.49- 1.14) | 0.181 |
| Fluctuating | 1002 (14.3) | 1.32 (1.01- 1.73) | 0.046**^*^** | 1.27 (0.97- 1.68) | 0.083 | 1.25 (0.95- 1.65) | 0.106 | 1.23 (0.93- 1.62) | 0.139 |
| Increasing | 581 (8.3) | 1.18 (0.84- 1.67) | 0.337 | 1.14 (0.80- 1.61) | 0.466 | 1.13 (0.80- 1.60) | 0.492 | 1.10 (0.77- 1.56) | 0.602 |
| Consistently high | 215 (3.1) | 1.87 (1.21- 2.91) | 0.005**^**^** | 1.81 (1.15- 2.83) | 0.010**^*^** | 1.70 (1.08- 2.67) | 0.022**^*^** | 1.62 (1.03- 2.56) | 0.037**^*^** |
| *Somatic trajectory of depressive symptom* | | | | | | | | | |
| Consistently low | 4475 (63.8) | Reference |  | Reference |  | Reference |  | Reference |  |
| Decreasing | 677 (9.6) | 1.33 (0.95- 1.87) | 0.098 | 1.32 (0.94- 1.86) | 0.112 | 1.29 (0.92- 1.82) | 0.142 | 1.23 (0.87- 1.74) | 0.243 |
| Fluctuating | 1014 (14.5) | 1.32 (0.99- 1.75) | 0.057 | 1.31 (0.98- 1.74) | 0.066 | 1.30 (0.97- 1.73) | 0.078 | 1.25 (0.93- 1.67) | 0.137 |
| Increasing | 618 (8.8) | 1.64 (1.21- 2.23) | 0.002**^**^** | 1.61 (1.18- 2.19) | 0.003**^**^** | 1.61 (1.18- 2.21) | 0.003**^**^** | 1.56 (1.14- 2.14) | 0.006**^**^** |
| Consistently high | 230 (3.3) | 1.80 (1.13- 2.89) | 0.014**^*^** | 1.76 (1.09- 2.84) | 0.021**^*^** | 1.75 (1.08- 2.85) | 0.024**^*^** | 1.63 (0.99- 2.66) | 0.053 |

**^†^**Model 1 adjusts for age only. **^‡^**Model 2 additionally adjusts for sociodemographics (sex, race, education, marital status, and income). **^§^** Model 3 additionally adjusts for health behaviors (body mass index, alcohol consumption, smoking status, and vigorous exercise). **^¶^**Model 4 additionally adjusts for health conditions (hypertension, heart condition, diabetes, stroke, lung disease, arthritis, and cancer). Significant associations are indicated by asterisks (**^*^***P* < 0.05; **^**^***P* < 0.01).

**Supplementary Table 3.** Cox proportional hazard ratios using stricter definitions for decreasing and increasing trajectory groups

|  |  | **Model 1 ^†^** |  | **Model 2 ^‡^** |  | **Model 3 ^§^** |  | **Model 4 ^¶^** |  |
| --- | --- | --- | --- | --- | --- | --- | --- | --- | --- |
|  | **No. of cases (%)** | **HR (95% CI)** | ***P*** | **HR (95% CI)** | ***P*** | **HR (95% CI)** | ***P*** | **HR (95% CI)** | ***P*** |
| *Total depressive symptom trajectory* | | | | | | | | | |
| Consistently low | 4412 (63.0) | Reference |  | Reference |  | Reference |  | Reference |  |
| Decreasing | 508 (7.2) | 0.87 (0.56- 1.35) | 0.531 | 0.85 (0.54- 1.32) | 0.467 | 0.86 (0.55- 1.34) | 0.501 | 0.84 (0.54- 1.30) | 0.426 |
| Fluctuating | 1462 (20.8) | 1.23 (0.96- 1.58) | 0.100 | 1.21 (0.94- 1.55) | 0.142 | 1.20 (0.93- 1.55) | 0.155 | 1.18 (0.92- 1.53) | 0.190 |
| Increasing | 331 (4.7) | 1.60 (1.08- 2.36) | 0.018**^*^** | 1.54 (1.04- 2.29) | 0.031**^*^** | 1.54 (1.04- 2.28) | 0.033**^*^** | 1.51 (1.01- 2.24) | 0.042**^*^** |
| Consistently high | 301 (4.3) | 1.81 (1.22- 2.69) | 0.003**^**^** | 1.79 (1.19- 2.68) | 0.005**^**^** | 1.72 (1.14- 2.60) | 0.009**^**^** | 1.70 (1.13- 2.57) | 0.012**^*^** |
| *Cognitive-affective trajectory of depressive symptom* | | | | | | | | | |
| Consistently low | 4617 (65.8) | Reference |  | Reference |  | Reference |  | Reference |  |
| Decreasing | 503 (7.2) | 0.87 (0.56- 1.35) | 0.529 | 0.84 (0.54- 1.30) | 0.429 | 0.83 (0.54- 1.30) | 0.423 | 0.82 (0.52- 1.27) | 0.367 |
| Fluctuating | 1387 (19.8) | 1.24 (0.97- 1.58) | 0.086 | 1.20 (0.93- 1.54) | 0.154 | 1.18 (0.92- 1.51) | 0.198 | 1.17 (0.91- 1.50) | 0.216 |
| Increasing | 292 (4.2) | 1.17 (0.74- 1.85) | 0.512 | 1.12 (0.71- 1.78) | 0.630 | 1.12 (0.70- 1.78) | 0.633 | 1.09 (0.69- 1.74) | 0.705 |
| Consistently high | 215 (3.0) | 1.87 (1.21- 2.91) | 0.005**^**^** | 1.83 (1.17- 2.87) | 0.008**^**^** | 1.73 (1.10- 2.72) | 0.018**^*^** | 1.71 (1.09- 2.69) | 0.020**^*^** |
| *Somatic trajectory of depressive symptom* | | | | | | | | | |
| Consistently low | 4475 | Reference |  | Reference |  | Reference |  | Reference |  |
| Decreasing | 574 | 1.15 (0.78- 1.70) | 0.482 | 1.15 (0.78- 1.70) | 0.481 | 1.13 (0.76- 1.68) | 0.538 | 1.11 (0.75- 1.65) | 0.600 |
| Fluctuating | 1402 | 1.43 (1.12- 1.83) | 0.004**^**^** | 1.42 (1.11- 1.82) | 0.005**^**^** | 1.42 (1.10- 1.82) | 0.006**^**^** | 1.39 (1.08- 1.78) | 0.010**^*^** |
| Increasing | 333 | 1.73 (1.17- 2.55) | 0.006**^**^** | 1.70 (1.15- 2.52) | 0.008**^**^** | 1.71 (1.15- 2.54) | 0.008**^**^** | 1.72 (1.16- 2.57) | 0.007**^**^** |
| Consistently high | 230 | 1.80 (1.13- 2.89) | 0.014**^*^** | 1.79 (1.11- 2.89) | 0.017**^*^** | 1.79 (1.10- 2.91) | 0.018**^*^** | 1.73 (1.06- 2.83) | 0.027**^*^** |

**^†^**Model 1 adjusts for age only. **^‡^**Model 2 additionally adjusts for sociodemographics (sex, race, education, and marital status). **^§^** Model 3 additionally adjusts for health behaviors (body mass index, alcohol consumption, smoking status, and vigorous exercise). **^¶^**Model 4 additionally adjusts for health conditions (hypertension, heart condition, diabetes, stroke, and lung disease). Significant associations are indicated by asterisks (**^*^***P* < 0.05; **^**^***P* < 0.01).

**Supplementary Table 4.** Hazard Ratios from Discrete-Time Models for the Association of Depressive Symptom Trajectories with Hip Fracture

|  |  | **Model 3 ^§^** |  | **Model 4 ^¶^** |  |
| --- | --- | --- | --- | --- | --- |
|  | **No. of cases (%)** | **HR (95% CI)** | ***P*** | **HR (95% CI)** | ***P*** |
| *Total depressive symptom trajectory* | | | | | |
| Consistently low | 4412 (62.9) | Reference |  | Reference |  |
| Decreasing | 609 (8.7) | 0.84 (0.56- 1.27) | 0.414 | 0.80 (0.53- 1.21) | 0.292 |
| Fluctuating | 1079 (15.4) | 1.18 (0.89- 1.57) | 0.258 | 1.14 (0.85- 1.52) | 0.375 |
| Increasing | 613 (8.7) | 1.59 (1.16- 2.17) | 0.004**^**^** | 1.53 (1.12- 2.09) | 0.008**^**^** |
| Consistently high | 301 (4.3) | 1.77 (1.17- 2.68) | 0.007**^**^** | 1.65 (1.09- 2.51) | 0.018**^*^** |
| *Cognitive-affective trajectory of depressive symptom* | | | | | |
| Consistently low | 4617 (65.8) | Reference |  | Reference |  |
| Decreasing | 599 (8.5) | 0.79 (0.52- 1.20) | 0.271 | 0.76 (0.50- 1.16) | 0.204 |
| Fluctuating | 1002 (14.3) | 1.29 (0.98- 1.70) | 0.068 | 1.27 (0.97- 1.68) | 0.088 |
| Increasing | 581 (8.3) | 1.17 (0.82- 1.66) | 0.388 | 1.13 (0.80- 1.61) | 0.480 |
| Consistently high | 215 (3.1) | 1.79 (1.14- 2.83) | 0.012**^*^** | 1.69 (1.07- 2.68) | 0.024**^*^** |
| *Somatic trajectory of depressive symptom* | | | | | |
| Consistently low | 4475 (63.8) | Reference |  | Reference |  |
| Decreasing | 677 (9.6) | 1.32 (0.93- 1.86) | 0.115 | 1.25 (0.88- 1.77) | 0.209 |
| Fluctuating | 1014 (14.5) | 1.31 (0.98- 1.75) | 0.067 | 1.26 (0.94- 1.68) | 0.125 |
| Increasing | 618 (8.8) | 1.63 (1.19- 2.23) | 0.002**^**^** | 1.57 (1.15- 2.16) | 0.005**^**^** |
| Consistently high | 230 (3.3) | 1.87 (1.15- 3.04) | 0.012**^*^** | 1.71 (1.04- 2.80) | 0.033**^*^** |

**^§^** Model 3 adjusts for sociodemographics (age, sex, race, education, and marital status) and health behaviors (body mass index, alcohol consumption, smoking status, and vigorous exercise). **^¶^** Model 4 additionally adjusts for health conditions (hypertension, heart condition, diabetes, stroke, and lung disease). Significant associations are indicated by asterisks (**^*^***P* < 0.05; **^**^***P* < 0.01).

**Supplementary Table 5.** Odds Ratios from Logistic Regression Models for the Association of Depressive Symptom Trajectories with Hip Fracture

|  |  | **Model 3 ^§^** |  | **Model 4 ^¶^** |  |
| --- | --- | --- | --- | --- | --- |
|  | **No. of cases (%)** | **OR (95% CI)** | ***P*** | **OR (95% CI)** | ***P*** |
| *Total depressive symptom trajectory* | | | | | |
| Consistently low | 4412 (62.9) | Reference |  | Reference |  |
| Decreasing | 609 (8.7) | 0.87 (0.56- 1.33) | 0.506 | 0.84 (0.54- 1.29) | 0.417 |
| Fluctuating | 1079 (15.4) | 1.20 (0.89- 1.62) | 0.240 | 1.19 (0.88- 1.60) | 0.266 |
| Increasing | 613 (8.7) | 1.65 (1.19- 2.29) | 0.003**^**^** | 1.62 (1.17- 2.26) | 0.004**^**^** |
| Consistently high | 301 (4.3) | 1.88 (1.21- 2.93) | 0.005**^**^** | 1.85 (1.19- 2.88) | 0.006**^**^** |
| *Cognitive-affective trajectory of depressive symptom* | | | | | |
| Consistently low | 4617 (65.8) | Reference |  | Reference |  |
| Decreasing | 599 (8.5) | 0.80 (0.52- 1.24) | 0.318 | 0.79 (0.51- 1.22) | 0.284 |
| Fluctuating | 1002 (14.3) | 1.34 (1.01- 1.79) | 0.045**^*^** | 1.34 (1.00- 1.78) | 0.049**^*^** |
| Increasing | 581 (8.3) | 1.20 (0.83- 1.73) | 0.331 | 1.19 (0.82- 1.71) | 0.364 |
| Consistently high | 215 (3.1) | 1.90 (1.17- 3.10) | 0.010**^*^** | 1.87 (1.15- 3.06) | 0.012**^*^** |
| *Somatic trajectory of depressive symptom* | | | | | |
| Consistently low | 4475 (63.8) | Reference |  | Reference |  |
| Decreasing | 677 (9.6) | 1.36 (0.95- 1.95) | 0.096 | 1.33 (0.92- 1.90) | 0.128 |
| Fluctuating | 1014 (14.5) | 1.33 (0.98- 1.80) | 0.064 | 1.31 (0.97- 1.77) | 0.083 |
| Increasing | 618 (8.8) | 1.70 (1.22- 2.37) | 0.002**^**^** | 1.69 (1.21- 2.35) | 0.002**^**^** |
| Consistently high | 230 (3.3) | 1.92 (1.14- 3.21) | 0.013**^*^** | 1.86 (1.10- 3.12) | 0.020**^*^** |

**^§^** Model 3 adjusts for sociodemographics (age, sex, race, education, and marital status) and health behaviors (body mass index, alcohol consumption, smoking status, and vigorous exercise). **^¶^** Model 4 additionally adjusts for health conditions (hypertension, heart condition, diabetes, stroke, and lung disease). Significant associations are indicated by asterisks (**^*^***P* < 0.05; **^**^***P* < 0.01).

**Supplementary Table 6.** Baseline characteristics of the study population stratified by inclusion status (Attrition Analysis)

| **Characteristics** | **Overall (N = 32087)** | **Excluded / Attrition (N = 25073)** | **Included in Analysis (N = 7014)** | ***P*-value** |
| --- | --- | --- | --- | --- |
| Age, y, mean (SD) | 66.2 (10.4) | 66.7 (11.3) | 64.4 (6.2) | <0.001 |
| *Sex, n (%)* |  |  |  | <0.001 |
| Female | 18024 (56.2) | 13732 (54.8) | 4292 (61.2) |  |
| Male | 14063 (43.8) | 11341 (45.2) | 2722 (38.8) |  |
| *Race, n (%)* |  |  |  | <0.001 |
| White | 28139 (87.7) | 21837 (87.1) | 6302 (89.8) |  |
| Non-white | 3948 (12.3) | 3236 (12.9) | 712 (10.2) |  |
| *Highest degree in education, n (%)* |  |  |  | <0.001 |
| High school and below | 21522 (67.1) | 17291 (69.0) | 4231 (60.3) |  |
| Some college | 5838 (18.2) | 4392 (17.5) | 1446 (20.6) |  |
| College and above | 4727 (14.7) | 3390 (13.5) | 1337 (19.1) |  |
| *Marital status, n (%)* |  |  |  | <0.001 |
| Married or partnered | 21418 (66.7) | 16363 (65.3) | 5055 (72.1) |  |
| Separated/ Divorced/ Widowed/ Never married | 10669 (33.3) | 8710 (34.7) | 1959 (27.9) |  |
| BMI, kg/m^2^, mean (SD) | 27.1 (5.0) | 26.9 (5.1) | 27.4 (4.7) | <0.001 |
| Hypertension (yes/no), n (%) | 13180 (41.1) | 10681 (42.6) | 2499 (35.6) | <0.001 |
| Diabetes (yes/no), n (%) | 3603 (11.2) | 3102 (12.4) | 501 (7.1) | <0.001 |
| Baseline CES-D, mean (SD) | 1.6 (2.0) | 1.7 (2.0) | 1.3 (1.8) | <0.001 |

**Supplementary Table 7.** Subdistribution Hazard Ratios (sdHR) from Fine-Gray Models for the Association Between Depressive Symptom Trajectories and Hip Fracture, Accounting for Death as a Competing Risk

|  |  |  | **Model 1 ^†^** |  | **Model 2 ^‡^** |  | **Model 3 ^§^** |  | **Model 4 ^¶^** |  |
| --- | --- | --- | --- | --- | --- | --- | --- | --- | --- | --- |
|  | **No. of cases (%)** | **Death, n (%)** | **sdHR (95% CI)** | ***P*** | **sdHR (95% CI)** | ***P*** | **sdHR (95% CI)** | ***P*** | **sdHR (95% CI)** | ***P*** |
| *Total depressive symptom trajectory* | | | | | | | | | | |
| Consistently low | 8664 (57.7) | 2062 (23.8) | Reference |  | Reference |  | Reference |  | Reference |  |
| Decreasing | 1414 (9.4) | 425 (30.1) | 0.99 (0.74- 1.34) | 0.963 | 0.94 (0.70- 1.26) | 0.672 | 0.95 (0.70- 1.27) | 0.716 | 0.95 (0.71- 1.29) | 0.755 |
| Fluctuating | 2506 (16.7) | 783 (31.2) | 1.14 (0.92- 1.43) | 0.237 | 1.08 (0.87- 1.35) | 0.475 | 1.09 (0.87- 1.36) | 0.455 | 1.10 (0.88- 1.37) | 0.407 |
| Increasing | 1606 (10.7) | 643 (40.0) | 1.50 (1.19- 1.89) | 0.001**^**^** | 1.43 (1.13- 1.80) | 0.003**^**^** | 1.43 (1.13- 1.81) | 0.003**^**^** | 1.45 (1.15- 1.84) | 0.002**^**^** |
| Consistently high | 829 (5.5) | 352 (42.5) | 1.62 (1.20- 2.18) | 0.001**^**^** | 1.49 (1.10- 2.01) | 0.009**^**^** | 1.51 (1.11- 2.04) | 0.008**^**^** | 1.55 (1.15- 2.11) | 0.005**^**^** |
| *Cognitive-affective trajectory of depressive symptom* | | | | | | | | | | |
| Consistently low | 9152 (60.9) | 2255 (24.6) | Reference |  | Reference |  | Reference |  | Reference |  |
| Decreasing | 1386 (9.2) | 413 (29.8) | 0.82 (0.59- 1.13) | 0.219 | 0.77 (0.56- 1.07) | 0.117 | 0.77 (0.56- 1.07) | 0.119 | 0.77 (0.56- 1.07) | 0.121 |
| Fluctuating | 2409 (16.0) | 796 (33.0) | 1.22 (0.99- 1.52) | 0.064 | 1.15 (0.93- 1.43) | 0.194 | 1.15 (0.93- 1.43) | 0.209 | 1.15 (0.93- 1.43) | 0.197 |
| Increasing | 1497 (10.0) | 569 (38.0) | 1.44 (1.14- 1.83) | 0.003**^**^** | 1.36 (1.08- 1.73) | 0.010**^*^** | 1.36 (1.07- 1.73) | 0.012**^*^** | 1.38 (1.08- 1.75) | 0.009**^**^** |
| Consistently high | 575 (3.8) | 232 (40.4) | 1.68 (1.19- 2.36) | 0.003**^**^** | 1.54 (1.09- 2.17) | 0.015**^*^** | 1.53 (1.08- 2.16) | 0.017**^*^** | 1.55 (1.10- 2.20) | 0.013**^*^** |
| *Somatic trajectory of depressive symptom* | | | | | | | | | | |
| Consistently low | 8738 (58.2) | 2008 (23.0) | Reference |  | Reference |  | Reference |  | Reference |  |
| Decreasing | 1551 (10.3) | 474 (30.6) | 1.26 (0.97- 1.63) | 0.078 | 1.20 (0.93- 1.56) | 0.166 | 1.21 (0.93- 1.57) | 0.154 | 1.23 (0.95- 1.60) | 0.117 |
| Fluctuating | 2418 (16.1) | 813 (33.6) | 1.26 (1.02- 1.57) | 0.033**^*^** | 1.23 (0.99- 1.52) | 0.064 | 1.24 (1.00- 1.54) | 0.055 | 1.27 (1.02- 1.59) | 0.034**^*^** |
| Increasing | 1655 (11.0) | 690 (41.7) | 1.20 (0.94- 1.54) | 0.146 | 1.16 (0.90- 1.48) | 0.247 | 1.17 (0.91- 1.51) | 0.217 | 1.19 (0.92- 1.53) | 0.179 |
| Consistently high | 657 (4.4) | 280 (42.6) | 1.67 (1.19- 2.33) | 0.003**^**^** | 1.55 (1.11- 2.17) | 0.010**^*^** | 1.60 (1.14- 2.25) | 0.007**^**^** | 1.70 (1.21- 2.39) | 0.003**^**^** |

The sample size in the Fine-Gray competing risk analysis differs from that in the primary Cox analysis because this sensitivity analysis retained participants with ascertainable hip fracture or death status during follow-up, including those without subsequent follow-up after hip fracture. sdHR, subdistribution hazard ratio; CI, confidence interval. **^†^**Model 1 adjusts for age only. **^‡^**Model 2 additionally adjusts for sociodemographics (sex, race, education, and marital status). **^§^** Model 3 additionally adjusts for health behaviors (body mass index, alcohol consumption, smoking status, and vigorous exercise). **^¶^**Model 4 additionally adjusts for health conditions (hypertension, heart condition, diabetes, stroke, and lung disease). Significant associations are indicated by asterisks (**^*^***P* < 0.05; **^**^***P* < 0.01).

**Supplementary Table 8.** Sex-stratified Fine-Gray competing risk analysis of the association between total depressive symptom trajectories and hip fracture risk

| **Depressive Symptom Trajectory** | **Female Subgroup** |  | **Male Subgroup** |  | ***P* for Interaction** |
| --- | --- | --- | --- | --- | --- |
|  | **sdHR (95% CI)** | ***P*** | **sdHR (95% CI)** | ***P*** |  |
| Consistently low | Reference |  | Reference |  | — |
| Decreasing | 0.92 (0.58- 1.46) | 0.725 | 0.54 (0.19- 1.51) | 0.238 | 0.327 |
| Fluctuating | 1.32 (0.95- 1.82) | 0.097 | 0.64 (0.32- 1.30) | 0.218 | 0.076 |
| Increasing | 1.57 (1.09- 2.24) | 0.015 | 1.48 (0.77- 2.81) | 0.233 | 0.994 |
| Consistently high | 1.55 (0.95- 2.50) | 0.076 | 2.55 (1.14- 5.74) | 0.024 | 0.206 |

The table presents subdistribution hazard ratios (sdHRs) and 95% confidence intervals (CIs) for the association between total depressive symptom trajectories and incident hip fracture, stratified by sex. Death before hip fracture was treated as a competing event. The consistently low trajectory served as the reference group. Models were adjusted for age, race, education, marital status, body mass index, alcohol consumption, smoking status, vigorous exercise, hypertension, heart condition, diabetes, stroke, and lung disease. The P for interaction was obtained by including a multiplicative interaction term between total depressive symptom trajectory and sex in the fully adjusted Fine-Gray subdistribution hazard model.

**Supplementary Table 9.** Age-stratified Fine-Gray competing risk analysis of the association between total depressive symptom trajectories and hip fracture risk

| **Depressive Symptom Trajectory** | **Age < 65 Subgroup** |  | **Age ≥ 65 Subgroup** |  | ***P* for Interaction** |
| --- | --- | --- | --- | --- | --- |
|  | **sdHR (95% CI)** | ***P*** | **sdHR (95% CI)** | ***P*** |  |
| Consistently low | Reference |  | Reference |  | — |
| Decreasing | 1.09 (0.56- 2.09) | 0.801 | 0.68 (0.40- 1.17) | 0.162 | 0.022 |
| Fluctuating | 1.26 (0.76- 2.08) | 0.363 | 1.08 (0.76- 1.54) | 0.680 | 0.499 |
| Increasing | 1.93 (1.12- 3.31) | 0.018 | 1.37 (0.94- 2.01) | 0.105 | 0.192 |
| Consistently high | 2.16 (1.05- 4.44) | 0.036 | 1.54 (0.92- 2.57) | 0.098 | 0.615 |

The table presents subdistribution hazard ratios (sdHRs) and 95% confidence intervals (CIs) for the association between total depressive symptom trajectories and incident hip fracture, stratified by age (<65 vs. ≥65 years). Death before hip fracture was treated as a competing event. The consistently low trajectory served as the reference group. Models were adjusted for sex, race, education, marital status, body mass index, alcohol consumption, smoking status, vigorous exercise, hypertension, heart condition, diabetes, stroke, and lung disease. The P for interaction was obtained by including a multiplicative interaction term between total depressive symptom trajectory and age group in the fully adjusted Fine-Gray subdistribution hazard model.
